# A perspective on “cure” for Rett syndrome

**DOI:** 10.1186/s13023-018-0786-6

**Published:** 2018-04-02

**Authors:** Angus John Clarke, Ana Paula Abdala Sheikh

**Affiliations:** 1 Institute of Medical Genetics, Division of Cancer & Genetics, School of Medicine, Cardiff University, Cardiff, Wales, UK; 2 School of Physiology, Pharmacology & Neuroscience, University of Bristol, Bristol, UK; 3 Institute of Medical Genetics, University Hospital of Wales, Heath Park, Cardiff, CF14 4XN Wales, UK

**Keywords:** *MECP*2, Rett syndrome, Cure, Expectations, Gene therapy, Gene editing, Symptomatic treatment, Quality of life

## Abstract

The reversal of the Rett syndrome disease process in the Mecp2 mouse model of Guy et al. (2007) has motivated families and researchers to work on this condition. The reversibility in adult mice suggests that there is potentially much to be gained from rational treatments applied to patients of any age. However, it may be difficult to strike the right balance between enthusiasm on the one hand and realism on the other. One effect of this has been a fragmentation of the “Rett syndrome community” with some groups giving priority to work aimed at a cure while fewer resources are devoted to medical or therapy-based interventions to enhance the quality of life of affected patients or provide support for their families.

Several possible therapeutic approaches are under development that, it is claimed and hoped, may lead to a “cure” for patients with Rett syndrome. While all have a rationale, there are potential obstacles to each being both safe and effective. Furthermore, any strategy that succeeded in restoring normal levels of *MECP2* gene expression throughout the brain carries potential pitfalls, so that it will be of crucial importance to introduce any clinical trials of such therapies with great care.

Expectations of families for a radical, rational treatment should not be inflated beyond a cautious optimism. This is particularly because affected patients with us now may not be able to reap the full benefits of a “cure”. Thus, interventions aimed at enhancing the quality of life of affected patients should not be forgone and their importance should not be minimised.

## Introduction

The road to a cure for Rett syndrome is paved with encouraging findings but also with many challenges. Whilst optimism drives discovery, it is important for those directly affected to be well informed. Scientists are all well aware that gene therapy and gene editing are still in their infancy. Setbacks during drug development are almost a certainty. However, this is not such common knowledge among the families of those affected. There is not enough information available to lay persons to allow realistic expectations to be set for the ‘cure’. No wonder the public becomes despondent and distrustful of experts when those inevitable ‘bumps-on-the-road’ arise, especially during clinical trials.

One consequence, perhaps, has been the polarisation of the Rett syndrome community. Some believe all efforts should be concentrated on finding a cure, instead of seeking new ameliorative or palliative treatments. Fewer now focus on care for the patients and families who remain in need. Choosing to support a cure-directed or a care-directed organisation may not be simply a matter of preference but is likely to relate to the stage of the condition in the affected individual and their pattern of adjustment to the diagnosis. The parents of more recently diagnosed children, who may indeed be better placed to benefit from new, cure-directed treatments, will often have more energy for fund-raising and more hope for a transformative treatment for their child. Parents of older patients will often be more focused on coping and caring, and will have adjusted to a different pattern of family life. This has meant that care-directed support groups have fewer resources for the continuing needs of the families they support.

This fragmentation of the Rett community can be seen as the result of complex social processes, including the rise of social media and the internet, and the rise of ‘the expert patient’. But, do we really want to place all our eggs in the single basket of transformative, cure-directed treatments?

## What we know about MECP2 and Rett syndrome

What we thought we knew about Rett syndrome before the genetic cause was discovered was only partially correct. Yet, the things that we got wrong turned out to be very interesting. For instance, *MECP2* mutations are not lethal in utero in males. They are simply more common in females. This is because de novo mutation in the *MECP2* gene happens more frequently in the production of sperm than of eggs, and therefore on the X chromosome donated by the father [1, 2]. In females, one of the two X chromosomes comes from her father, whereas in males the X always comes from the mother and the Y chromosome from the father. Accordingly, the mutation is found much more commonly in females. De novo mutations in the paternal germline are more common than previously thought. We now know that the risk of mutations in sperm increases with exposure to environmental toxins and with age [3].

It has also been learned that Rett syndrome appears to be a neurodevelopmental disorder because of a “necessary coincidence”. This is because the person who carries the mutation is, of course, deficient in the MECP2 protein from the time they begin to grow and develop. However, in a study that switched off production of the protein in adult mice, the researchers showed the same range of symptoms as those seen in mice which had the mutation from conception and had therefore developed with the disease [4]. It is likely that the same clinical picture would happen in adult humans if the gene were to switch off later in life. This is good news because most true neurodevelopmental disorders are not reversible in adulthood. In contrast, the study by Guy J, Gan J, Selfridge J, Cobb S and Bird A [5] showed that it is possible to reverse the symptoms of Rett syndrome in affected adult mice. While those experiments did not provide a clear route to develop human therapies, they did provide an enormous motivator. These experiments indicated that the potential benefits from any effective treatments could be significant no matter the patient’s age. Furthermore, both studies also highlighted that any type of gene therapy will need to deliver a working *MECP2* gene throughout the person’s life. It is not enough to deliver it just during the child’s development.

There is some relation between the type of mutation and the severity of certain symptoms in Rett syndrome. However, this is inevitably obscured by variations of X chromosome inactivation. Females have two X-chromosomes, which means that they could have double the amount of X-linked gene products compared to males. But this does not happen, because one X-chromosome is always (largely) inactive. In some females, who are carriers of *MECP2* mutations, the X chromosome carrying the mutation can be preferentially inactivated in some or most cells [6]. This often makes their symptoms milder or even absent and they do not develop Rett syndrome. This variability in X chromosome inactivation - frustratingly - makes it harder to distinguish the genetic from non-genetic modifiers of disease severity. But it also provides an alternative route for a cure (*more on this below*).

## Challenges for gene therapy

One of the greatest known challenges to delivering a permanent, “for ever”, cure for Rett syndrome comes from what scientists call the ‘Goldilocks principle’. That is, the amount of protein needs to be just right in each brain cell, as too much MECP2 protein can be as bad as too little. We know this because of another condition called *MECP2* duplication syndrome [7]. The males affected by this condition have two copies of the *MECP2* gene on their single X-chromosome, and so their cells produce too much protein. The disease is also characterised by mental disability and autistic-like behaviour. Even worse, the levels of MECP2 do not need to be much different for brain function to be disrupted. Smaller deviations in protein function have been linked with milder neurological and psychiatric symptoms. For instance, female carriers of *MECP2* duplication in which 85% or more cells inactivate the X chromosome carrying the duplication (so that the duplication is functional in < 15% of cells) exhibit anxiety and depression [8, 9]. Others with less favourable inactivation bias, have additional intellectual disability and Rett-like symptoms [9, 10]. This potentially means that to really cure a person with Rett syndrome we would need to deliver the right levels of MECP2 to nearly all brain cells. Additionally, in females, we would need to avoid delivering additional copies of the gene to cells that already express the healthy copy. This is a major challenge within the constraints of the currently available technologies. We may have to accept that we can improve most of the severe symptoms but not all. The person with Rett undergoing gene therapy might still have underlying psychiatric symptoms due to the sub-optimal levels of MECP2 in parts of their brain.

Hopes of overcoming the ‘Goldilocks’ issue are based on the process of X-chromosome inactivation. A treatment that manages to inactivate the X chromosome carrying the damaged copy of the *MECP2* gene could resume production of functional MECP2 protein from the intact copy on the other X chromosome. This is an attractive treatment option as cells would use their own regulatory functions to produce just the right amount of MECP2 protein. We do not know how or why some people are able to skew their X-chromosome inactivation, although the tendency to do this can run in families and there might be damaging but unforeseen consequences in some individuals. Of course, it will sometimes happen simply by chance. Another approach might be to activate the inactivated copy of the *MECP2* gene in every cell. For this to work, however, reactivation would need to be very selective to that single gene. We do not yet have the technology to achieve this and, again, there might be unintended consequences. Even if we could do this, this approach would not offer a solution for all people with Rett syndrome. Those carrying mutations that leave some residual MECP2 activity would end up with too much protein function.

Gene editing is a powerful new technology that could correct most of the *MECP2* mutations causing Rett syndrome. It could overcome the ‘Goldilocks’ issue and would work in both males and females. Gene editing uses molecular scissors to search a specific DNA sequence and remove it. The faulty sequence is cut out and replaced with the corrected DNA code. These molecular scissors are produced in nature by bacteria. They use it to remove harmful viral genes from their genome. This technology, called CRISPR/Cas9, is already widely used to produce genetically altered animals for research [11]. It recently made the news when Ma H, Marti-Gutierrez N, Park SW, Wu J, Lee Y, Suzuki K, Koski A, Ji D, Hayama T, Ahmed R, Darby H, Van Dyken C, Li Y, Kang E, Park AR, Kim D, Kim ST, Gong J, Gu Y, Xu X, Battaglia D, Krieg SA, Lee DM, Wu DH, Wolf DP, Heitner SB, Belmonte JCI, Amato P, Kim JS, Kaul S and Mitalipov S [12] successfully used it to correct a gene mutation in human embryos. However, this technique will not be approved for use in humans for some time, and the embryos used in that research were destroyed. The method worked relatively well for fertilised eggs before they had developed into embryos. Other studies have shown that this approach is less effective once the fertilised eggs have developed into embryos [12]. This has a potential application to preventing an inherited disorder being transmitted by an affected parent but its application to the treatment of affected patients appears much more difficult. This would be especially true for a condition such as Rett syndrome where the key effects of the disease result from problems in the brain. Previous studies in mice have shown that the technology is sometimes not sufficiently precise and that it causes mutations in unintended sites [13]. Even a very low level of incorrect DNA targeting could cause disease in the individual patient being treated, especially cancers. Mutations may also be triggered that introduce recessive disease gene mutations, unlikely to affect the treated patient but potentially causing a range of different diseases in future generations. Clearly, further refinement and safety testing will be required. Some suggest that a complete genome sequencing of each patient would be required. This is to ensure that the CRISPR/Cas9 would not target unwanted sites of the patient’s genome. Related technological approaches that do not modify DNA but RNA raise similar difficulties but would in addition require life-long administration. These approaches, therefore, may be less suitable as a therapeutic strategy.

Finally, like other gene therapy strategies, there are technical challenges with delivering the gene editing constructs into the brain [14].

## Perils of a late cure

Whichever strategy is chosen, there is no doubt that some form of treatment for Rett syndrome will be available soon. Until now, 40 trials for Rett syndrome have been registered in ClinicalTrials.gov. This is a registry of publicly and privately supported clinical studies conducted around the world. Most involve modulators of neurotransmission, which aim to mitigate specific symptoms of the disease. These are symptomatic treatments, not cures, as they don’t tackle the root cause, i.e. the disruption to the *MECP2* gene that results in deficiency of the MECP2 protein. Charities are moving away from funding these types of studies. With costs of drug development being so high, it would seem logical to invest heavily in strategies for curing or reversing the disease, instead of treatments that merely moderate symptoms without ‘correcting’ the underlying problem. But could a ‘cure’ have unintended hazardous effects, be incomplete or contraindicated for some?

Based on animal experiments, this appears to be a warranted concern. The first set of adult mice to be ‘cured’ by Guy et al. [5] suffered high mortality. Nine out of the first seventeen mice to be treated, all males, died soon after treatment. The first treatment was given over just 5 days. Survival was not problematic when the reactivation of *Mecp2* proceeded more slowly. The timescale of the mortality suggests that this may have been a result of irregular breathing or erratic control of the heart rhythm, although we do not know that for certain. Certainly, it would be no surprise if autonomic instability were to accompany any effective intervention in the expression of the *MECP2* gene (or the *Mecp2* gene in mice).

Furthermore, the re-expression of *MECP2* in people with Rett syndrome is likely to increase the volume of their brains. We know that people with Rett syndrome have smaller head circumference than neurotypical individuals. Thus, curing Rett might be problematic in young people and adults whose skull sutures have fused. There will be no room for the brain to grow. Cranial surgery may be required to prevent brain damage or fatalities. It is the autonomic instability and raised intracranial pressure that we might expect to be hazardous over the days or weeks following an attempted ‘cure’ and which would need to be monitored closely.

We must also not underestimate the sensory and psychological impacts of being ‘cured’. The experience of regression early in the disease course causes very evident distress. In individuals being ‘cured’, the process of re-emerging from the cocoon imposed by the disease might not be trouble-free. Some brain functions that will re-awaken might be unpleasant, for instance pain. People with Rett syndrome are reported to have reduced sensitivity to pain [15]. Treated individuals may begin to experience greater than usual pain. Given the difficulties with communication, it is expected that strategies of pain assessment and management will need to be in place for those receiving any attempt at a ‘cure’. Some of these difficulties may be temporary but not all. Unfortunately, for some young people and adults, the musculoskeletal deformities caused by Rett will be inexorably irreversible. These individuals might end up requiring life-long pain management therapies.

It is also worth considering the impact of an incompletely effective rational therapy on behaviour. The benefits are evident: any small improvement in cognition, in control of breathing and speech, in ability to walk, in ability to use hands would be welcomed by patients and carers. However, affected individuals could become physically fitter and abler while still having cognitive and communication deficits, which may be associated with psychological distress and confusion brought on by treatment-mediated changes. Such individuals, may start to display very challenging behaviours and become more difficult to care for than before treatment. It is expected that strategies would be put in place to help relatives and carers manage challenging behaviours, whether temporary or permanent, that may arise from an incomplete cure.

## Conclusions

By now you are probably asking yourself if you should even hope for a cure. We would reply with a very definite, “Yes”, but also with some caution. Toddlers and younger children with Rett have a very good chance of experiencing a major improvement from new types of treatment and even, perhaps, a cure. For those further through childhood, the answer may be a middle ground where we hope for the best but prepare for the worst. If there is one lesson that the history of therapeutic discoveries has taught us, it is that throwing money at the problem is no guarantee of the outcome you hoped for. While that shouldn’t stop us from seeking a cure, perhaps we should not give up the search for therapies that bring benefit without necessarily achieving a cure. After all, a child with Rett syndrome now will probably be a young adult by the time “the cure” has first been developed and then made readily available.

## Glossary

Cas9: short for CRISPR associated protein 9. A genetic engineering tool used to cut a specific DNA sequence. It is usually associated with a CRISPR sequence.
CRISPR: abbreviation for Clustered Regularly Interspaced Short Palindromic Repeats. A genetic engineering tool used to locate a specific DNA sequence.
*De novo* mutations: a genetic alteration that is present for the first time in one family member because of a mutation in a germ line cell of one of the parents, or that arises in the fertilized egg itself during early development.
DNA: Deoxyribonucleic acid, the molecule that stores the material of heredity in cells within the body and and transmits it to future generations.
et al.: and the others
Genome: the complete set of genes or genetic material present in an organism
Germ line: cells that during reproduction pass on their genetic material to the offspring. These include the gametes, sperm and egg, and the cells from which they were produced.
MECP2 (italicised): the gene that encodes methyl-CpG-binding protein 2 (MECP2). Mutations in this gene cause Rett syndrome.
MECP2 (non-italicised): a protein that controls the expression of other genes. This protein appears to be essential for the normal function of brain cells.
MECP2 (all letters in capitals): is the version of the gene or protein in humans, Mecp2 (only the first letter in capitala) is the version in the mouse.
RNA: Ribonucleic acid, a material related to DNA. It has multiple roles in the cell, especially in the synthesis of proteins. This includes acting as a messenger molecule that conveys the information in DNA from the cell nucleus into the cytoplasm so that it can be translated into protein.
X chromosome: a sex chromosome, two of which are normally present in female cells (designated XX) and only one in male cells (designated XY).
